# Synthesis of Silver Nanoparticles from Extracts of Wild Ginger (*Zingiber zerumbet*) with Antibacterial Activity against Selective Multidrug Resistant Oral Bacteria

**DOI:** 10.3390/molecules27062007

**Published:** 2022-03-21

**Authors:** Muhammad Ramzan, Mohmed Isaqali Karobari, Artak Heboyan, Roshan Noor Mohamed, Mohammed Mustafa, Syed Nahid Basheer, Vijay Desai, Salma Batool, Naveed Ahmed, Basit Zeshan

**Affiliations:** 1 Faculty of Life Sciences, University of Central Punjab, Lahore 54000, Punjab, Pakistan; ramzan.qarshi82@gmail.com (M.R.); salma.batool@ucp.edu.pk (S.B.); 2 Saveetha Dental College, Saveetha Institute of Medical and Technical Sciences, Saveetha University, Chennai 600077, Tamil Nadu, India; 3 Department of Restorative Dentistry & Endodontics, Faculty of Dentistry, University of Puthisastra, Phnom Penh 12211, Cambodia; 4 Department of Prosthodontics, Faculty of Stomatology, Yerevan State Medical University after Mkhitar Heratsi, Str. Koryun 2, Yerevan 0025, Armenia; 5 Department of Pediatric Dentistry, Faculty of Dentistry, Taif University, P.O. Box 11099, Taif 21944, Saudi Arabia; roshan.noor@tudent.edu.sa; 6 Department of Conservative Dental Sciences, College of Dentistry, Prince Sattam Bin Abdulaziz University, P.O. Box 173, Al-Kharj 11942, Saudi Arabia; ma.mustafa@psau.edu.sa; 7 Department of Restorative Dental Sciences, Division of Operative Dentistry, College of Dentistry, Jazan University, Jazan 45142, Saudi Arabia; snbasheer@jazanu.edu.sa; 8 College of Dentistry, Ajman University, Al Jurf, Ajman P.O. Box 346, United Arab Emirates; v.desai@ajman.ac.ae; 9 Department of Medical Microbiology and Parasitology, School of Medical Sciences, University Sains Malaysia, Kubang Kerian, Kota Bharu 16150, Kelantan, Malaysia; namalik288@gmail.com; 10 Faculty of Sustainable Agriculture, University Malaysia Sabah, Sandakan Campus, Locked Bag No. 3, Sandakan 90509, Sabah, Malaysia

**Keywords:** biosynthesized silver nanoparticles, AgNPs, MDR pathogens, wild ginger extract, antibacterial activity

## Abstract

Antibiotic resistance rate is rising worldwide. Silver nanoparticles (AgNPs) are potent for fighting antimicrobial resistance (AMR), independently or synergistically. The purpose of this study was to prepare AgNPs using wild ginger extracts and to evaluate the antibacterial efficacy of these AgNPs against multidrug-resistant (MDR) *Staphylococcus aureus*, *Streptococcus mutans*, and *Enterococcus faecalis*. AgNPs were synthesized using wild ginger extracts at room temperature through different parameters for optimization, i.e., pH and variable molar concentration. Synthesis of AgNPs was confirmed by UV/visible spectroscopy and further characterized by scanning electron microscopy (SEM), X-ray diffraction (XRD), energy-dispersive X-ray spectroscopy analysis (EDXA), and Fourier-transform infrared spectroscopy (FTIR). Disc and agar well diffusion techniques were utilized to determine the in vitro antibacterial activity of plant extracts and AgNPs. The surface plasmon resonance peaks in absorption spectra for silver suspension showed the absorption maxima in the range of 400–420 nm. Functional biomolecules such as N–H, C–H, O–H, C–O, and C–O–C were present in *Zingiber zerumbet* (*Z. zerumbet*) (aqueous and organic extracts) responsible for the AgNP formation characterized by FTIR. The crystalline structure of ZZAE-AgCl-NPs and ZZEE-AgCl-NPs was displayed in the XRD analysis. SEM analysis revealed the surface morphology. The EDXA analysis also confirmed the element of silver. It was revealed that AgNPs were seemingly spherical in morphology. The biosynthesized AgNPs exhibited complete antibacterial activity against the tested MDR bacterial strains. This study indicates that AgNPs of wild ginger extracts exhibit potent antibacterial activity against MDR bacterial strains.

## 1. Introduction

The significance of nanotechnology lies in its vast industrial applications in biomedical and bioengineering fields [1]. In recent decades, nanoparticles (NPs) have been recognized as one of the most cutting-edge materials. Many procedures are involved in the synthesis of NPs, such as heat evaporation [2], chemical reduction [3], biological [4], electrochemical [5], reverse micelle, thermal decomposition, radiation, and microwave-assisted strategies [6]. Most of the techniques are time-consuming and require different hazardous chemicals to produce NPs.

The utilization of plants for the synthesis of NPs has an advantage as compared to other natural strategies (for example, microorganisms) because it does not require a long cultivation time to reduce the metal particles. Plant-made NPs are cost-effective and have nontoxic impacts [7]. Different factors such as temperature, duration, and pH influence the characteristics of the plant-mediated NPs [8]. The plant extract acts as a bio-reductant agent and stabilizes the operator amid the arrangement of AgNPs due to bioactive constituents such as alkaloids, flavonoids, volatile oils, and phenolic compounds [9]. Plants are utilized as the source of potential chemotherapeutic and antimicrobial agents in ethnomedicine.

Metabolites or phytochemicals produced from plants, herbs, spices, and fruits are natural products. Plant bioactive compounds are an alternate technique for reducing the prevalence of infectious pathogens. In the pharmaceutical industry, plant-based substances are considered a preliminary base for drug research and production [10]. The “antibiotic era” began in the mid-20th century, and it shifted the attention away from natural product-based medicine. However, the rise of AMR strains and various adverse effects of synthetic chemotherapeutics have again sparked interest in looking for biocompatible natural antimicrobial agents [11,12].

Recently, pathogenic bacteria such as *Staphylococcus aureus* (*S. aureus*), *Klebsiella pneumoniae* (*K. pneumoniae*), *Escherichia coli* (*E. coli*), *Salmonella* spp., and *Pseudomonas* spp. illustrated resistance to commercially accessible antimicrobial drugs at an expanding rate worldwide, particularly in developing countries. The presence of multidrug-resistant (MDR) pathogens has expanded the cases of infectious diseases and become the leading cause of mortality worldwide [13]. Extensive misuse and abuse of antibiotics are driving cases of AMR within the microbes [14]. In recent times, the utilization of sullied poultry eggs and their products are known to be the most common source of food-borne infections [15]. The emergence of AMR strains of *S. aureus*, *Enterococcus faecalis* (*E. faecalis*), and *Streptococcus mutans* (*S. mutans*) is a worldwide challenge for clinical pharmaceuticals. The impact of *S. aureus*, *E. faecalis*, and *S. mutans* incorporates many skin problems, gastrointestinal issues, urinary tract infections, oral infections, and numerous other infections. AgNPs demonstrated compelling antibacterial efficacy against MDR strains of *K. pneumoniae*, *E. coli*, *E. faecalis*, *S. mutans*, and *S. aureus* in previous studies [12,16,17].

The World Health Organization (WHO) believes that 80% population of the world still uses traditional medicine, including plant-based medications [4]. One of the most utilized herbs in folk medicine is ginger. It is used as a spice and flavoring agent worldwide, and it is said to have a variety of medical benefits [18]. Many research studies have discovered its many pharmacological effects, including antioxidant, antibacterial, anti-inflammatory, antinociceptive, antimutagenic, and hepatoprotective activities [10,19]. Zingiberaceae is one of the most prominent families of the plant world and is most often used as raw material to produce various traditional medicines [20]. *Z. zerumbet*, commonly known as wild ginger, is a member of this family has many bioactive compounds which play an essential role as reducing and capping agents. Therefore, it would be of high interest to take advantage of them for the green synthesis of AgNPs. Clinical and nourishment experts profoundly prescribe wild ginger rhizomes for use in traditional cures and flavors, as well as for the treatment of health complaints. Currently, different antimicrobial agents are being developed for use on bacterial infections which are resistant to antibiotics. In addition to the development of new antibiotics, there is a need for alternate ways of treating various bacterial infections in order to overcome the high mortality rates because of high AMR among bacteria. Keeping in mind the situation, the current study provides data on the synthesis of the AgNPs using wild ginger extracts. In the current study, wild ginger rhizome extracts were initially utilized for the biosynthesis of NPs. The green biosynthesis of AgNPs was characterized by various physical procedures such as UV/Vis, followed by XRD, FTIR, and SEM–EDXA. The antimicrobial activity of aqueous and organic wild ginger extracts was determined against *S. aureus*, *Streptococcus mutans*, and *Enterococcus faecalis*. Green-synthesized AgNPs were also evaluated against the MDR bacterial species.

## 2. Results

### 2.1. The Phytochemical Analysis of Plant

The phytochemical ingredients responsible for AgNP reduction and capping in aqueous and organic extracts of *Z. zerumbet* were investigated qualitatively. The phytochemical screening of *Z. zerumbet* (aqueous and organic) extracts indicated a high content of secondary metabolites, as shown in Table 1.

### 2.2. Optimization of Silver Nanoparticles

For the ideal biogenic synthesis of AgNPs, different tests varying parameters such as the pH of the reaction mixture, the concentration of the silver nitrate solution, and the incubation time were performed. UV/Vis spectra were recorded to evaluate the changes as a result of varying the response parameters, thus identifying their optimal values.

### 2.3. Effect of pH on ZZ-AgNP Synthesis

**Figure 1:**
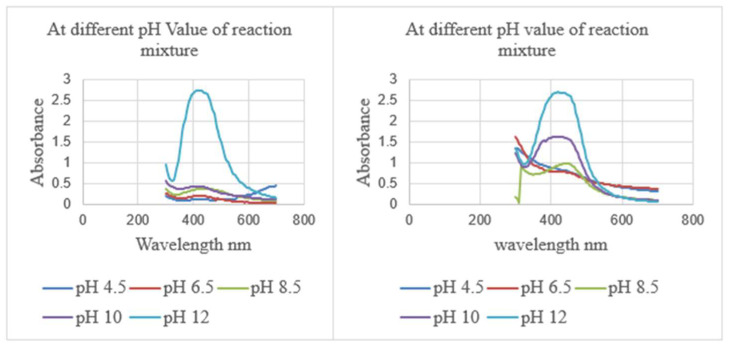
UV/Vis spectra (**Left**) upon varying pH using a 10 mM AgNO_3_ aqueous solution (125 mL) and ZZAE (25 mL) reaction mixture at ambient temperature for 24 h and (**Right**) upon varying pH using a 10 mM AgNO_3_ aqueous solution (125 mL) and ZZEE (25) reaction mixture at room temperature for 24 h.

The impact of pH was considered the most important parameter influencing the NP arrangement. Figure 1 shows the effect of varying pH on NP biosynthesis according to UV/visible spectra (UV/Vis) absorption bands when producing AgNPs using *Z. zerumbet* (aqueous and organic extracts). The volume (25 mL) of organic and aqueous plant extract, concentration (10 mM) of aqueous AgNO_3_ solution, the incubation time (24 h), and the temperature (room temperature) were kept constant. Nanoparticles were observed to be well synthesized at a basic pH of 12, which is excellent. The intensity of plasmon absorbance bands was increased when pH increased from 8.5 to 12, but there were few plasmon absorbance bands at acidic pH levels of 6.5 to 4.5 (Figure S1).

It was also observed that, after mixing aqueous silver nitrate solution with both extracts at pH 6.5 to pH 12, the color changed from brown to yellowish-brown or deep yellow after 24 h. UV/Vis spectra were recorded 24 h after the incubation period upon varying the pH of the NP suspension, as shown in Figure 1.

### 2.4. Effect of Reaction Time on ZZ-AgNP Synthesis

**Figure 2:**
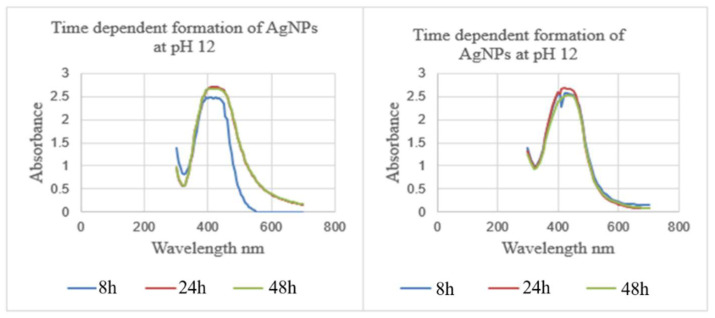
UV/Vis spectra (**Left**) of the time-dependent formation of AgNPs at pH 12 using a 10 mM AgNO_3_ aqueous solution (125 mL) and ZZAE (25 mL) reaction mixture at room temperature at different intervals and (**Right**) of the time-dependent formation of AgNPs at pH 12 using a 10 mM AgNO_3_ aqueous solution (125 mL) and ZZEE (25 mL) reaction mixture at room temperature at different intervals.

For the synthesis of AgNPs, reaction time is also considered an essential factor. Reaction time is the period for the complete reduction of silver ions for the synthesis of AgNPs. To study the effect of incubation time, the volume (25 mL) of organic and aqueous plant extract, the concentration (10 mM) of aqueous AgNO_3_ solution, the pH (12) of the reaction mixture, and the temperature (room temperature) were kept constant. The influence of incubation time on the biosynthesis of NPs was evaluated according to the UV/Vis absorption bands of AgNPs synthesized using *Z. zerumbet* (aqueous and organic extracts) (Figure 2). The absorption band was observed from 400–420 nm at different incubation times, as shown in Figure 2. The peak intensity gradually increased with time and decreased after 48 h. NPs were found to be stable after 24 h in the NP suspension. Hence, the UV/Vis study confirmed that silver ions were reduced to AgNPs using *Z. zerumbet* (aqueous and organic extracts).

### 2.5. Effect of Silver Nitrate Concentration

**Figure 3:**
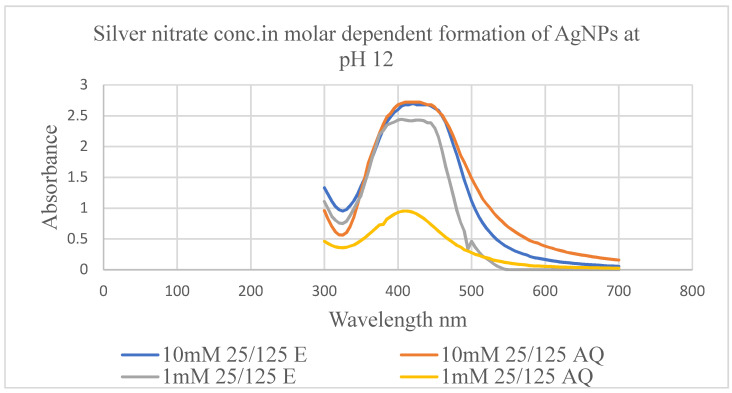
Molarity-dependent formation of AgNPs at pH12 using varying molar concentrations of the AgNO_3_ aqueous solution and plant extract (ZZAE and ZZEE) (1 mL of plant extract and 5 mL of silver nitrate solution) reaction mixture at room temperature for 24 h.

To consider the impact of silver nitrate concentration, the volume (25 mL) of organic and aqueous plant extracts, pH (12) of the reaction mixture, incubation time (24 h), and temperature (room temperature) were kept constant. The impact of silver nitrate concentration on the biosynthesis of AgNPs was evaluated according to UV/Vis absorption bands of AgNPs synthesized using *Z. zerumbet* rhizome extract (aqueous and organic extract) (Figure 3). The creation of AgNPs was indicated by a distinctive surface plasmon resonance (SPR) band peak in the visible range from 400 to 500 nm. This peak did not appear in the UV/Vis spectrum of silver nitrate solution or *Z. zerumbet* aqueous and organic extracts. While examining the effect of 125 mL of the prepared 1 mM and 10 mM silver nitrate concentrations on AgNPs synthesis, a high absorbance peak was discovered to be associated with a high concentration of silver nitrate, indicating increased AgNP organization (Figure 3 and Table 2). After a 24 h incubation period at pH 12 in a reaction mixture with different silver nitrate concentrations, the UV/Vis spectra of AgNPs were observed. According to the results, a 10 mM concentration of silver nitrate was ideal for further research.

### 2.6. XRD Analysis

**Figure 4:**
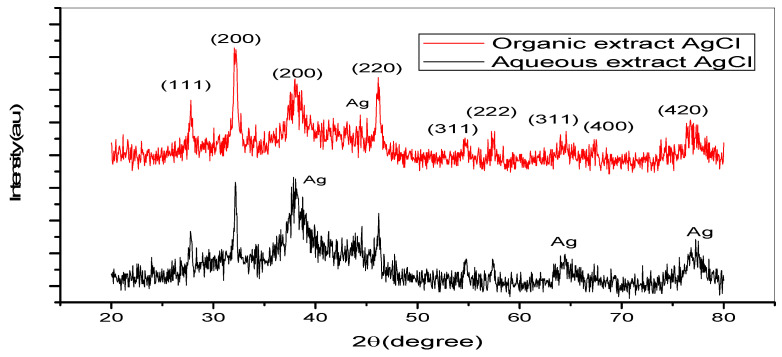
XRD pattern image of synthesized silver nanoparticles using organic and aqueous extract of *Z. zerubmet*.

The XRD patterns for AgNPs generated using the organic and aqueous extracts of *Z. zerumbet* rhizome are shown in Figure 4. The peaks at the 2θ values ranged from 20° to 80°. The peaks of NPs at 27.80°, 32.25°, 38.12°, 46.13°, 54.64°, 57.15°, 64.26°, 67.46°, and 76.87° in the organic extract of the *Z. zerumbet* rhizome and 27.80°, 32.15°, 34.21°, 37.92°, 43.83°, 46.13°, 54.76°, 57.45°, 64.26°, 67.46°, and 77.07° in the aqueous extract of the *Z. zerumbet* rhizome were indexed as crystalline silver. The crystalline peaks of AgNPs in the XRD pattern were 38.12°, 37.92°, 43.83°, 64.26°, and 77.07°, which can be indexed to the face-centered cubic structure (111, 111, 200, 220, and 311 planes, respectively). The XRD pattern revealed the presence of the face-centered cubic phase of silver chloride at 2θ values of 27.80°, 32.15°, 46.13°, 54.76°, 57.45°, 67.46°, and 77.87°, corresponding to the 111, 200, 220, 311, 222, 400, and 420 planes, respectively. We used the highest-intensity peak of silver chloride to compute the average crystalline particle size of silver chloride nanoparticles. The 200 and 220 lattice planes of organic silver chloride extract and aqueous silver chloride extract were chosen. Using Debye–Scherer’s equation, the average crystalline particle size was calculated to be 26.65 nm for the organic extract of silver chloride and 21.3 nm for the aqueous extract of silver chloride.

(1) $$D=\frac{K\lambda }{\beta cos\theta },$$

where *D* is the average crystalline size, *K* is a dimensionless shape factor, with a value close to unity (0.99), *λ* is the wavelength of Cu-Kα, *β* is the full width at half maximum of the diffraction peaks, and *θ* is Bragg’s angle.

### 2.7. SEM and EDX Analysis

**Figure 5:**
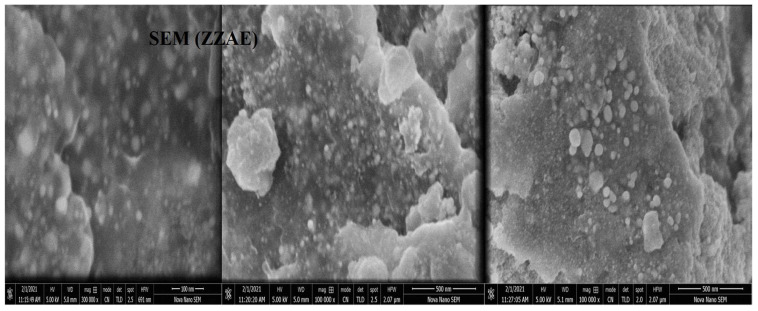
SEM micrograph showing the shape of AgNPs synthesized from ZZAE at different magnifications.

**Figure 6:**
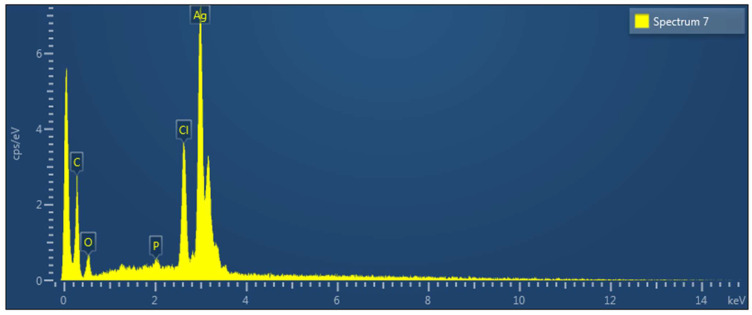
EDX spectra demonstrating the quantitative amounts of different elements in the silver nanoparticles synthesized using an aqueous extract of *Z. zerumbet*.

To characterize the morphology of synthesized NPs, SEM was used. The SEM images in Figure 5 clearly show the creation of a spherical shape. The EDXA study revealed a peak for silver at 3 keV, as well as various peaks for C, O, P, and Cl, all of which came from biomolecules bound to the surface of AgNPs, as shown in Figure 6 (Figures S2 and S3).

### 2.8. FTIR Spectroscopy Analysis

Figure S5 represents the ATR-FTIR spectra of *Z. zerumbet* extract, as well as its resultant ZZ-Ag-NPs. Because of reduction, capping, and stabilization of the synthesized NPs, the ATR-FTIR spectra of *Z. zerumbet* (aqueous and organic) extracts and AgNPs (manufactured using the aqueous and organic extracts) displayed substantial and minor shifts. Because of O–H or N–H stretching of phenolics in *Z. zerumbet* rhizome (aqueous and organic) extracts, the peak at 3355.93 cm^−1^ and 3271.70 cm^−1^ shifted to a lower wavelength (3206.12 cm^−1^ and 3177.30 cm^−1^, respectively). The absorption peaks characteristic of terpenoids and saponins at 2910.64 cm^−1^, 2911.30 cm^−1^, 2914.85 cm^−1^, 2968.82 cm^−1^, 2839.65 cm^−1^, and 2841.68 cm^−1^ were attributed to C–H stretching in a methylene group or an aliphatic group. A shift was also identified for the peak at 1567.54 cm^−1^ with a higher wavelength of 1619.80 cm^−1^, as well as for the peak at 1699.59 cm^−1^ with a lower wavelength of 1622 cm^−1^, due to the participation of alkenyl or aromatic C=C stretching. The FTIR spectrum in the range 1650–1550 cm^−1^ represents C=C, C=N, N–H stretching, while the range 1550–1300 cm^−1^ represents NO_2_ or CH_3_ and CH_2_ stretching. The peak range 1300–1000 cm^−1^ shows the presence of C–O–C, C–OH, and P=O functional groups in the sample. The peaks in the range 1000–650 cm^−1^ are relevant to =C–H and N–H stretching, while the peaks in the range 800–500 cm^−1^ suggest the presence of halogen compounds and aromatic compounds. The majority of peaks indicated the presence of phenolic compounds, steroids, tannins, flavonoids, terpenoids, alkaloids, and saponins, all of which are abundant in the *Z. zerumbet* rhizome (aqueous and organic) extracts and aided in the synthesis of AgNPs. The phytochemical study of *Z. zerumbet* rhizome (aqueous and organic) extracts confirmed these findings.

### 2.9. DLS and Zeta Potential Analysis

#### Zeta Potential and Size Measurement

**Figure 7:**
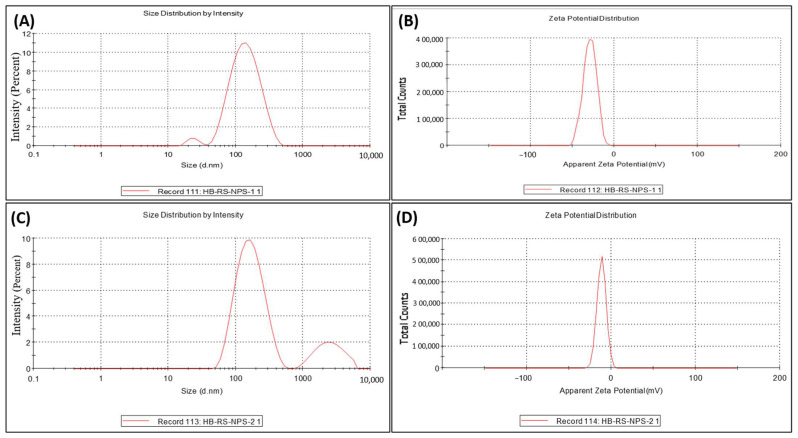
(**A**) DLS pattern of AgNPs biosynthesized using ZZEE; (**B**) zeta potential measurement of AgNPs biosynthesized using ZZEE; (**C**) DLS pattern of AgNPs biosynthesized using ZZAE; (**D**) zeta potential measurement of AgNPs biosynthesized using ZZAE.

The size and zeta potential of biosynthesized AgNPs using *Z. zerumbet* at pH 12 (aqueous and organic extracts) were determined using a Zeta Sizer Nano-ZS (Figure 7A–D). Figure 7A shows the DLS pattern of AgNPs biosynthesized using ZZEE. The particle size distribution result showed two peaks at 153.2 nm (96.9%) and 24.18 nm (3.1%). The zeta potential of AgNPs synthesized using *Z. zerumbet* organic and aqueous extracts was measured to be −29.0 and −11.5 mV (Figure 7B,D), respectively. The negative charge on NPs was due to the presence of the functional groups of wild ginger extract. In addition, the FTIR spectra of synthesized NPs showed the involvement of different functional groups responsible for the stabilization of NPs, which acted as capping and stabilizing agents.

### 2.10. Antimicrobial Susceptibility Test (Commercial Antibiotics)

The antibiotic sensitivity testing of *Streptococcus mutans* and *Enterococcus faecalis* strains revealed resistance to amoxicillin (Ax25), azocillin (Az75), cloxacillin (Cx1), oxacillin (Ox1), and ticarcillin (Ti75). *S. aureus* showed resistance to amoxicillin (Ax25), azocillin (Az 75), cloxacillin (Cx1), oxacillin (Ox1), and ticarcillin (Ti75). *S. aureus* showed sensitivity to amoxicillin (Ax25) with a clear zone of inhibition (10 mm). The sensitivity pattern of tested organisms against each antibiotic is shown in Table S1.

#### 2.10.1. Antibacterial Assay of AgNPs Synthesized at pH 12 Using Plant Extract against MDR *S. Aureus*, *Streptococcus mutans*, and *Enterococcus faecalis*

The abovementioned organisms showed significant zones of inhibition after antibiotic testing with the AgNPs synthesized at pH = 12 using plant extracts. The zones of inhibition after the well agar disc diffusion method are shown in Table 3.

#### 2.10.2. Minimum Inhibitory Concentration (MIC) Determination of Synthesized ZZEE-Ag-NPs and ZZAE-Ag-NPs against MDR Bacteria

After 24 h of incubation at 37 °C, the micro-titer plates of AgNPs synthesized using organic extracts of *Z. zerumbet* (ZZEE-AgNPs) showed no inhibition of MDR *S. aureus*, *Streptococcus mutans*, and *Enterococcus faecalis* in rows F, G, and H. On the other hand, rows A, B, and C has showed MICs of ZZEE-AgNPs against the tested bacteria with optical densities of 0.327, 0.123, and 0.003, respectively. The MIC (µg/mL) values of synthesized ZZAE-Ag-NPs and ZZAE-Ag-NPs against the three isolated MDR strains of *S. aureus*, *S. mutans*, and *E. faecalis* are shown in in Table 4 (Figure S4).

## 3. Discussion

The biological production of NPs is an emerging approach for treatment and diagnosis, with advantages of using nontoxic bioactive molecules (found in plant extract), being cost-effective, and involving single-step strategies for their assembly, thus attracting researchers and clinicians [21]. Because of their small size, AgNPs have specific physical and chemical properties. NPs have a more significant bactericidal effect than bulk metallic silver because of the reduction in size, increase in the surface-to-volume ratio, and increase in the contact area with microorganisms [22]. The main objectives of the present study were to investigate the synthesis of AgNPs using wild ginger extracts and to check their antibacterial activity.

The addition of aqueous and organic extracts of *Z. zerumbet* rhizome to a silver nitrate solution (10 mM) produced a color change from colorless to pale yellow. The addition of sodium hydroxide (NaOH) in green synthesis has previously been reported by Singh et al. [23]. After 24 h of the conversion process, AgNPs displayed a yellow-brown (for aqueous extract) or deep yellow (for the organic extract) color, suggesting the formation of AgNPs in the reaction mixture solution. These results are consistent with previous studies [18,24,25]. In the current study, the creation of AgNPs was also verified using a UV/Vis spectrophotometer at wavelengths between 300 and 700 nm. The excitation of SPR vibration in NPs caused absorbance bands at wavelength 419 nm (for the organic extract) and 407.5 nm (for the aqueous extract) at pH 12, indicating the formation of AgNPs and reduction of silver ions by the active compounds present in wild ginger rhizome extracts. In previous studies, it was stated that collective electron oscillation around the particle’s surface mode is an important property in the optical absorbance of metal NPs in the surface plasmon band [21,26,27]. In another study, the absorption spectra of generated AgNPs showed a single surface plasmon resonance band, indicating their spherical shape [4]. The results of the current study reveal that the surface plasmon band expanded with the increase in silver nitrate concentration. Previously, this was seen in cases of olive leaf extract [28] and *Megarphyrynium marcrostachym* leaf extract [29], indicating optimization of the process and the production of more NPs.

Wild ginger (aqueous and organic extract) acts as a capping agent and a green reductant, allowing for the creation and stability of AgNPs with plasmon resonance bands in the 400–420 nm range [30]. The plasmon absorbance band was also diminished at low pH and strengthened at higher pH. According to studies using *Megarphyrynium marcrostachym* leaf extract and *Selaginella myosurus* aqueous extract, the plasmon absorbance band increased with pH from 4.5 to 12 [29]. This is due to the ability of the reaction pH to change the electrical charges of biomolecules, potentially affecting their capping and stabilizing capabilities and, as a consequence, the production of NPs [31]. Previous studies demonstrated that changing the pH of the reaction mixture may change the size and form of biosynthesized NPs by enhancing the pace of the reduction reaction [30], as shown in olive leaf extract and *Pinus eldarica* bark extract [4]. The number of NPs produced and their stability are affected by pH. It is an essential factor regulating the size and shape of NPs.

The XRD result in the current study indicated the production of Ag/AgCl composite NPs using aqueous and organic extracts of *Z. zerumbet*. The formation of AgCl-NPs can be attributed to the interaction of silver ions with silver chloride ions present in the aqueous and organic extracts of *Z. zerumbet*. Similar results were previously reported regarding the biosynthesis of AgNPs [32]. In the current study, the formation of crystalline AgNPs was also confirmed by XRD analysis. Kumar and Yadav observed a similar result to our study [33]. Furthermore, Jeeva et al. [34] identified peaks of 32.28°, 46.28°, 67.47°, and 76.69°.

DLS depends on the interaction of light with particles, and it can be utilized for the measurements of molecule dissemination, particularly within the range 2–500 nm [35]. In a previous study, the AgNPs were bigger according to DLS compared to XRD [36]. This difference can be clarified by the fact that the size measured by DLS is based on the combination of particles and the hydrodynamic radius, which is not a genuine estimate of the Ag-NP size due to the hydration layer around the particles, as well as the presence of capping and stabilizing operators [36]. In the current study, the particle size distribution showed two peaks at 153.2 nm (96.9%) and 24.18 nm (3.1%).

The polydispersity index (PDI) measures the homogeneity of NPs. It is essentially a representation of the distribution of size populations inside a given test sample. The numerical value of PDI ranges from 0.0 to 1.0. NPs with a PDI value < 0.3 are considered satisfactory for sedate conveyance [36]. The synthesized AgNPs had a normal PDI of 0.237, indicating their homogeneity and potential effectiveness in different applications [37]. In the present study, the zeta potential of AgNPs synthesized using *Z. zerumbet* organic and aqueous extracts was measured to be −29.0 and −11.5 mV, respectively.

The SEM–EDXA result confirmed that the AgNPs were spherical in shape. The aggregation of NPs indicates that they were in direct contact but stabilized by a capping agent. Functional groups had a stable size and were responsible for capping of AgNPs. The presence of elemental silver was verified by the EDXA signal at 3 keV. The emission energy of 3 keV indicated the reduction of silver ion to elemental silver. Metallic silver nanocrystals usually show strong absorption spectra in the range 2.5–3.5 keV. Similar findings were reported in many previous studies [34,38,39].

MDR bacterial strains have been reported worldwide, posing a severe threat to public health [40]. In the current study, three clinically isolated strains of bacteria, *S. aureus*, *S. mutans*, and *E. faecalis*, were tested for AMR using the disc diffusion method, and their susceptibility to the biosynthesized AgNPs and wild ginger extracts was evaluated, as summarized in Table 3. These bacteria showed resistance to the tested antibiotics using the disc diffusion method. However, in the presence of the synthesized AgNPs and wild ginger extracts, the antibacterial activity was seen in the form of clear zones of inhibition. The antibacterial susceptibilities of MDR strains of different bacteria using ginger extracts were previously reported in different studies [11,41,42], including *S. aureus* [10]. It could be possible that the Ag component of AgNPs confers antimicrobial properties. A strong reaction takes place between the silver ions and thiol groups of vital enzymes, ultimately inactivating them.

The present study indicates the importance of synthesized AgNPs in overcoming MDR *S. aureus*, *Streptococcus mutans*, and *E. faecalis* strains. The green-synthesized AgNPs showed significant antibacterial activities against clinically isolated MDR *S. aureus*, *Streptococcus mutans*, and *E. faecalis* strains. AgNPs were shown to have broad-spectrum antibacterial action against various pathogenic bacteria, even including extensively drug resistant (XDR) bacteria, in previous studies [10,12]. AgNPs were previously described as effective bactericidal agents for killing microorganisms [22]. In another investigation, NPs made from *S. potatorum* leaf extract were shown to be effective against *S. aureus*, an MDR human pathogenic bacterium [43]. Similar observations were reported for *Bosewllia ovalifliolata* and *Shoera tumbuggai* [44]. AgNPs prepared from aqueous and organic extracts of *Z. zerumbet* showed antibacterial efficacy against MDR bacteria. Gram-positive bacterial cell walls have a dense peptidoglycan layer composed of linear polysaccharide chains crossed by short peptides, forming a stiffer framework, which hinders the penetration of AgNPs made from aqueous and organic extracts of *Z. zerumbet*. In contrast, Gram-negative bacterial cell walls have a thin layer of peptidoglycan [42,43].

The MIC values from prior investigations differed widely. Consequently, comparing the results is challenging since there is no standard technique for determining AgNP antibacterial activity, and studies have used different methods [44]. The current study showed that the biosynthesized AgNPs have antimicrobial activity.

## 4. Materials and Methods

### 4.1. Plant Extracts

Fresh rhizomes of *Z. zerumbet* were collected in November and December 2020 from the Changa Manga Forest (Pakistan); the plant was identified using its vernacular name by the Department of Botany, University of Punjab, Quaid-I-Azam Campus, Lahore.

After collection, the rhizomes were washed thoroughly with distilled water, shade-dried, and identified by the Herbarium, Department of Botany, University of the Punjab, Lahore, where a voucher (LAH#280920) specimen of plant species was deposited for further reference. The shade-dried plant material was coarsely powdered in a grinder or mill and passed through the mesh no. five, before storing in an airtight container for further work (extraction).

### 4.2. Preparation of Aqueous Extract of Z. zerumbet

Ten grams of coarse powder of *Z. zerumbet* rhizome was taken in a glass bottle (size: 250 mL), and 100 mL of distilled water (1:10 *w*/*v*) was added and then mixed thoroughly. After mixing, the mixture was boiled at 35 °C for 30 min using a water bath and then allowed to cool at room temperature. After cooling, the mixture was filtered using Whatman filter paper and stored at 5 °C to be further used for the synthesis of AgNPs [31], while some filtrates were evaporated to dryness at 40 °C in a vacuum using a rotary evaporator. The extract was stored in an airtight container or universal glass bottle and kept at 5 °C in a refrigerator until further experiments for the crude extract’s biological activity and phytochemical analysis.

### 4.3. Preparation of Organic Extract of Z. zerumbet

Twenty-five grams of powder plant material was boiled with 250 mL of ethanol (99%) for 2 h (at 70 °C) using a Soxhlet apparatus and filtered using Whatman filter paper. After filtration, it was stored at 5 °C for further experiments to synthesize AgNPs; then, using a rotary evaporator in a vacuum-controlled environment, the filtrate was evaporated to dryness at 40 °C. The extract was stored in an airtight container or universal glass bottle and kept at 5 °C in a refrigerator until further experiments for the crude extract’s biological activity and phytochemical analysis.

### 4.4. Preliminary Phytochemical Analysis

A preliminary phytochemical investigation was conducted on extracts to determine the phytoconstituents present in *Z. zerumbet* rhizome (aqueous and organic extract) such as flavonoids, phenolics, terpenoids, alkaloids, and saponins, using the standard phytochemical procedure described by Cyril et al. [10].

### 4.5. Synthesis of NPs Using Z. zerumbet Aqueous and Organic Extract

Before starting the procedure, aqueous silver nitrate solutions (1 mM and 10 mM) were prepared. Spectrophotometric measurements based on color change were used to determine the formation of AgNPs. The AgNPs were synthesized according to previous procedures with slight modification [27,45]. The prepared (aqueous and organic) wild ginger extracts (25 mL) were gradually mixed with the prepared 1 mM silver nitrate aqueous solution (125 mL) at room temperature in a 500 mL glass bottle for the bio-reduction process. The pH (4.5, 6.5, 8.5, 10, and 12) of the mixtures in the 500 mL glass bottles was varied using HCl (0.1 M) and NaOH (0.1 M), determined by a pH meter. The silver nitrate solutions were incubated in a dark environment for 24–48 h to prevent photoactivation. The reduction of silver ions to AgNPs was observed preliminary on the basis of a color change to yellow or dark brown. The ZZ-AgNP formation was established by UV/Vis (HALO DB-20/DB-20S). The effect of aqueous and organic extracts of *Z. zerumbet*, pH of the reaction mixtures, incubation time, and silver nitrate concentration on the shape, size, and distribution of ZZ-Ag-NPs was studied. All AgNP biosynthesis experiments were conducted in batch mode.

#### 4.5.1. Effect of pH Change

The prepared (aqueous and organic) wild ginger extracts (25 mL) were gradually mixed with the prepared 1 mM silver nitrate aqueous solution (125 mL) at room temperature in a 500 mL glass bottle for the bio-reduction process. To pH of the reaction mixtures was changed using HCl (0.1 M) and NaOH (0.1 M), determined by a pH meter. The silver nitrate solutions were incubated in a dark environment for 24 h to prevent photo-activation. After incubating the AgNPs for the synthesis process, 1 mL of ginger extract/AgNO_3_ solution was taken. About 1 mL (diluted suspension with 1:20 *v*/*v* distilled water) of AgNP solution synthesized using *Z. zerumbet* was monitored by UV/Vis (HALO DB-20/DB-20S; 300–700 nm operated at a resolution of 0.5 nm) 24 h after the beginning of the reaction. The same procedure was then repeated for various pH values of the reaction mixture at 6.5, 8.5, 10, and 12.

#### 4.5.2. Effect of AgNO_3_ Concentration

Two concentrations were chosen as the effective concentration of silver nitrate (1 mM and 10 mM). The prepared (aqueous and organic) wild ginger extracts (25 mL) were gradually mixed with the prepared 1 mM silver nitrate aqueous solution (125 mL) at room temperature in a 500 mL glass bottle for the bio-reduction process, and the pH of the reaction mixture was changed to 12 using 0.1 M NaOH, determined by a pH meter. The silver nitrate solutions were incubated in a dark environment for 24 h to prevent photo-activation. After incubating the AgNPs for the synthesis process, 1 mL of ginger extract/AgNO_3_ solution was taken. About 1 mL (diluted suspension with 1:20 *v*/*v* distilled water) of AgNP solution synthesized using *Z. zerumbet* was monitored by a UV/Vis spectrophotometer (300–700 nm operated at a resolution of 0.5 nm) 24 h after the beginning of the reaction. The same procedure was then repeated for the 10 mM AgNO_3_ concentration.

#### 4.5.3. Centrifugation

The resulting brown or deep yellow solution was centrifuged at 6000 rpm for 20 min (Table Top Centrifuge, DIG-SYSTEM Laboratory Instruments Inc., New Taipei City, Taiwan) to remove the upper liquid phase, and the remaining pellets were washed twice with 5 mL of distilled water and once with 99% ethanol to get rid of the free proteins/enzymes that were not capping the silver nanoparticles. The purified pellets were moved to a Petri plate and placed in a hot air oven at 60 °C for 24 h for drying; they were used later for biochemical characterization and antimicrobial activities.

### 4.6. Characterization of AgNPs

#### 4.6.1. XRD Analysis

The XRD profile of biosynthesis AgNPs was obtained (powdered samples) by XRD using a Model D8 Advance Bruker, at 20°–80° using Cu Kα radiation at 40 kV and 30 mA [46].

#### 4.6.2. Scanning Electron Microscopy EDX Detector Analysis (SEM-EDXA)

SEM was used to examine the morphology of AgNPs synthesized using *Z. zerumbet* aqueous and organic extracts, which was operated at a 12 keV accelerating voltage. The sample was prepared by simply dropping a minimal sample on a carbon-coated copper grid. After drying for 5 min under a mercury lamp, SEM scans at different magnifications were obtained. The dried powdered AgNP sample was drop-coated on a carbon sheet for elemental analysis. After that, an EDX detector connected to the SEM was used to perform the analysis [47].

#### 4.6.3. ATR-FTIR Analysis

To determine the probable functional groups involved in the reduction and stabilization of AgNPs, an alpha Bruker model in transmittable mode was used to record ATR-FTIR spectra of the dried powder of *Z. zerumbet* crude extract; the subsequent results of synthesized AgNPs were recorded in the region of 4000–500 cm^−1^ at room temperature [46,47].

### 4.7. Determination of Particle Size and Zeta Potential

The size distribution pattern was assessed using the dynamic light scattering technique. The electric charge of the particles according to their zeta potential was measured using a Zeta Sizer Nano-ZS instrument (Malvern instrument LTD, ver. 7.10, Malvern, Worcestershire, UK) following the protocol by Siddiqi et al. (2018) [26]. In a falcon tube, the purified biosynthesized AgNPs were reconstituted in distilled water employing a mechanical shaker and subsequently sonicated in an ultrasonic bath for 10 min. An aliquot of 1 mL was taken and placed in polyethylene cuvettes for DLS and particle size determination in the Zeta Sizer with the following parameters: material refractive index 1.33, dispersant 1.330, material absorption 0.0001, and viscosity 0.8872 cP. The software was set to automatic acquisition mode. Hydrodynamic diameter was calculated utilizing the software’s tools.

### 4.8. Collection and Preparation of Bacterial Strains

For testing antibacterial activity, *S. aureus*, *Streptococcus mutans*, and *Enterococcus faecalis* strains were obtained from the Microbiology Laboratory, the University of Veterinary and Animal Sciences Lahore, Punjab. These bacterial strains were sub-cultured on blood agar, nutrient agar, and nutrient broth to obtain the fresh bacterial colonies. After inoculation, the plates were incubated at 37 °C for 24 h. After the incubation period, the bacterial colonies were identified using different biochemical tests including catalase, coagulase, DNA, and the bile esculin test [13,44].

### 4.9. Antibiotic Susceptibility of the Three Oral Pathogenic Strains

The antibacterial sensitivity of bacterial strains was determined by the Kirby–Bauer disc diffusion method following Wayne (2012) [48]. Using a sterile cotton swab, nutrient agar plates were swabbed with a fresh culture of bacterial isolates. The surface of the medium was allowed to dry for about 2 or 3 min. The commercial antibiotic discs included amoxicillin (25 µg), oxacillin, ticarcillin (75 µg), azocillin (75 µg), and cloxacillin. The antibiotic discs were placed on inoculated agar plates using sterile forceps and incubated at 37 °C for 24 h. After 18 to 24 h of incubation, the zone of inhibition was measured in mm using a measuring ruler, and the means of three measurements were determined. Any bacterial strains resistant to ≥3 antibiotics were defined as MDR pathogens [49].

#### 4.9.1. Agar Well Diffusion Method

The antibacterial activity of synthesized AgNPs (using aqueous and organic extracts of *Z. zerumbet*) and plant extract against MDR *S. aureus*, *Streptococcus mutans*, and *Enterococcus faecalis* was determined using the standard protocol described in Wayne (2012) [48]. Nutrient agar plates were swabbed with a fresh culture of pathogenic organisms using a sterile cotton swab. The surface of the medium was allowed to dry for about 2 or 3 min. The agar was then punched aseptically using sterile tips to make holes 5 mm in diameter. Fifty microliters of *Z. zerumbet* plant extract (100 µg of each plant extract was dissolved in 1 mL of PBS) and AgNPs synthesized using *Z. zerumbet* (100 µg of each biosynthesized AgNP sample was dissolved in 1 mL of PBS) were added to respective wells using a micropipette. Each treatment was replicated three times and incubated at 37 °C for 24 h. The zone of inhibition was measured in mm using a measuring ruler after 24 h of incubation, and the means of three measurements were calculated.

#### 4.9.2. Estimation of MICs for Synthesizing AgNPs

To characterize the antibacterial activity of the synthesized AgNPs using aqueous and organic extracts of *Z. zerumbet*, the broth microdilution method was performed according to the standard protocol described in Wayne (2012) [48] and Vassallo et al. [50]. *S. aureus*, *Streptococcus mutans*, and *Enterococcus faecalis* were grown in a nutrient broth (NB) medium. Concentrations of 50, 25, 12.5, 6.25, 1.56, 0.78.0.39, 0.195, and 0.097 µg/mL of AgNPs synthesized using aqueous and organic extracts of *Z. zerumbet* were used to evaluate their antibacterial effect on *S. aureus*, *Streptococcus mutans*, and *Enterococcus faecalis*. The broth microdilution technique was used to determine the minimum inhibitory concentration (MIC) in sterile 96 well micro-titer plates. Sterile nutrient broth (100 µL) was added to rows A, B, C, F, G, and H. Then, 100 µL of silver nanoparticles synthesized using organic extract of *Z. zerumbet* at a concentration of 100 µg/mL (100 µg of each sample of biosynthesized silver nanoparticles was dissolved in 1 mL of PBS) were added to column 1 or the first well of rows A, B, and C. Furthermore, 100 µL of AgNPs synthesized using aqueous extracts of *Z. zerumbet* at a concentration of 100 µg/mL were added to column 1 or the first well of rows F, G, and H of the plate micro-titer that contained 100 µL of PBS to obtain a concentration of 50 µg/mL AgNPs synthesized using organic and aqueous extracts of *Z. zerumbet*. Serial dilution was performed by removal of 100 µL from the first well or column 1 of rows A, B, C, F, G and H and adding to the second well. This operation was applied to 10 columns or wells of the respective rows. Then, 100 µL of bacterial suspension, equivalent to 0.5 McFarland (1 × 10^7^ CFU/mL inoculums) was added to each well of the respective rows except the 11th column, obtaining a final volume of 200 µL. The negative control (PBS and medium) was in column 11, while the positive control (medium and inoculums) was in column 12. The plates were incubated for 24 h at 37 °C. Using an ELISA microplate reader, the optical density (OD) of each well was measured at 630 nm before and after incubation for 24 h. The values of each well were calculated using the following formula:

Values = OD1 − OD2,(2)

where OD1 is the value measured after incubation, and OD2 is the value measured before incubation.

The abbreviations used in the current study are shown in Table S2.

## 5. Conclusions

Different antimicrobial compounds are being investigated by several researchers for use against bacteria and yeasts resistant to antibiotics. The synthesis of AgNPs was carried out in this investigation utilizing wild ginger extracts. UV/Vis, XRD, FTIR, and SEM–EDXA techniques were used to characterize the produced AgNPs. Using SEM, it was discovered that the AgNPs generated by wild ginger extracts had a spherical configuration. Green synthesis is a low-cost, straightforward, and environmentally friendly process, and the biosynthesis of AgNPs described herein is an alternative to chemical synthesis methods. On MDR bacterial strains, the biosynthesized AgNPs had a good antibacterial impact. Green-synthesized AgNPs will open up a new pharmaceutical sector for the manufacture of pharmaceutical, biomedical, and industrial goods.

## Figures and Tables

**Table 1:** The qualitative phytochemical screening of wild ginger extracts (aqueous and organic).

Test		ZZEE	ZZAE
Test for alkaloid	Wagner’s test	−	−
	Mayer’s test	−	−
Test for tannins and phenolic compound	Ferric chloride test	+	+
	Gelatin test	+	+
Test for saponin glycosides	Saponin test	+	+
Test for flavonoids glycoside	Alkaline reagent test	−	−
Test for phytosterols	Phytosterol test	−	−
Test for steroid and triterpenoids	Liebermann–Buchard’s test	+	+
	Salkowski test	+	+

ZZAE = *Z. zerumbet* aqueous extracts, ZZEE = *Z. zerumbet* organic extracts, + = positive, − = negative.

**Table 2:** Peak wavelength and absorbance of silver nanoparticles using aqueous and organic extracts of the dried rhizome of *Z. zerumbet*.

Concentration (mM)	pH	Organic Extract (ZZEE-AgNPs) Wavelength (nm)	Organic Extract (ZZEE-AgNPs) Absorbance	Aqueous Extract (ZZAE-Ag-NPs) Wavelength (nm)	Aqueous Extract (ZZAE-AgNPs) Absorbance
1	12	402.0, 413.0, and 420	2.443, 2.443, and 2.443	410.0	0.953
10		419.0	2.699	407.5	2.721

ZZEE-AgNPs = AgNPs synthesized using organic extract of *Z. zerumbet*; ZZAE-AgNPs = AgNPs synthesized using aqueous extract of *Z. zerumbet*.

**Table 3:** The zones of inhibition (mm) of AgNPs synthesized using aqueous and organic extracts of *Z. zerumbet*.

Sr. No.	MDR Bacterial Strains	Zone of Inhibition (mm) Means ± SD	
		ZZAE-Ag-NPs	ZZEE-Ag-NPs
1	*Enterococcus faecalis*	19.33 ± 0.57	19.83 ± 0.57
2	*Streptococcus mutans*	17.83 ± 0.57	18.83 ± 0.57
3	*S. aureus*	13.83 ± 0.57	14.66 ± 0.57

Note: Plant extracts (ZZAE and ZZEE) did not show antimicrobial activity against MDR pathogen strains. MDR multidrug-resistant, ZZAE-Ag-NPs = AgNPs synthesized using aqueous extract of *Z. zerumbet*, ZZEE-Ag-NPS = AgNPs synthesized using organic extract of *Z. zerumbet*, ZZAE = *Z. zerumbet* aqueous extract, ZZEE = *Z. zerumbet* ethanolic extract. (Sr.No = Serial Number and Means ± SD = Mean ± standard deviation).

**Table 4:** MIC (µg/mL) values of synthesized ZZAE-Ag-NPs and ZZEE-Ag-NPs against the isolated MDR *S. aureus*, *Streptococcus mutans*, and *Enterococcus faecalis*.

Row	AMR Bacterial Strains (Clinical Isolates)	MIC (µg/mL) Antimicrobial Activity	
		ZZEE-Ag-NPS (50 µg/mL)	ZZAE-Ag-NPs (50 µg/mL)
A	*S. aureus*	3.12	
B	*Enterococcus faecalis*	6.25	
C	*Streptococcus mutans*	12.5	
F	*S. aureus*	-	25
G	*Enterococcus faecalis*	-	6.25
H	*Streptococcus mutans*	-	25

Micro-titer plates showing MIC top three row of silver nanoparticles using aqueous extract and bottom three rows of Ag-NPs using organic extract. Top Three rows A–C: *Staphylococcus aureus*, *Enterococcus faecalis* and *Streptococcus mutans*, the bottom three rows F–H: *Staphylococcus aureus*, *Enterococcus faecalis* and *Streptococcus mutans*.

## Data Availability

The dataset used in the current study will be made available upon reasonable request.

## Supplementary Materials

The following supporting information can be downloaded at https://www.mdpi.com/article/10.3390/molecules27062007/s1: Figure S1. Visual observation of color changes upon varying pH after 24 h of silver nanoparticles biosynthesized using (A) aqueous extracts and (B) organic extracts of *Z. zerumbet*; Figure S2. SEM micrograph showing the shape of AgNPs synthesized from ZZEE at different magnifications; Figure S3. EDX spectra demonstrating the quantitative amounts of different elements in the silver nanoparticles synthesized using organic extract of *Z. zerumbet*; Figure S4. Micro-titer plates showing MICs of silver nanoparticles synthesized using aqueous extract in top three rows and AgNPs synthesized using organic extract in bottom three rows. A–C: *Staphylococcus aureus*, *Enterococcus faecalis*, and *Streptococcus mutans;* F–H: *Staphylococcus aureus*, *Enterococcus faecalis*, and *Streptococcus mutans*. Columns 1–10: twofold serial dilutions. Top three rows: 50 µg/mL ZZEE AgNPs; bottom three rows: 50 µg/mL ZZAE-Ag-NPs. Column 11: NG (negative control well). Column 12: PG (positive control well); Figure S5. ATR-FTIR spectra of (A) AgNPs biosynthesized using organic extract of *Z. zerumbet* and (B) AgNPs biosynthesized using aqueous extract of *Z. zerumbet*; Figure S6. Mechanism for silver ion reduction to metallic silver nanoparticles via quercetin as reducing agent in *Z. zerumbet* Rhizome extract solution; Figure S7. Schematic illustration of biosynthesized silver nanoparticle (ZZEE-Ag-NPs and ZZAE-Ag-NPs) Capped by phytoconstituents through carboxyl, hydroxyl and silver interactions; Figure S8. ATR-FTIR spectra of (A) ZZEE and resultant ZZEE-Ag-NPs and (B) ZZAE and resultant ZZAE-AG-NPs; Figure S9. SEM micrograph showing the shape of AgNPs synthesized from ZZAE and ZZEE at different magnifications; Figure S10. EDX spectra demonstrating the quantitative amounts of different elements in the silver nanoparticles synthesized using aqueous and organic extracts of *Z. zerumbet*; Table S1. Antibacterial efficacy of commercial antibiotics against *E. coli*, *Salmonella enterica*, and *S. aureus*; Table S2. Abbreviations and their definitions.

## Sample Availability

Samples of the compounds are not available from the authors.
