# Supplementary material for: Synthesis of Silver Nanoparticles from Extracts of Wild Ginger (Zingiber zerumbet) with Antibacterial Activity against Selective Multidrug Resistant Oral Bacteria
Source: Molecules. 2022 Mar 21;27(6):2007. doi: 10.3390/molecules27062007 (PMC8949094; doi:10.3390/molecules27062007)
Supplement: Supplementary file 1 [file molecules-27-02007-s001.zip › molecules-1634947-supplementary.pdf]

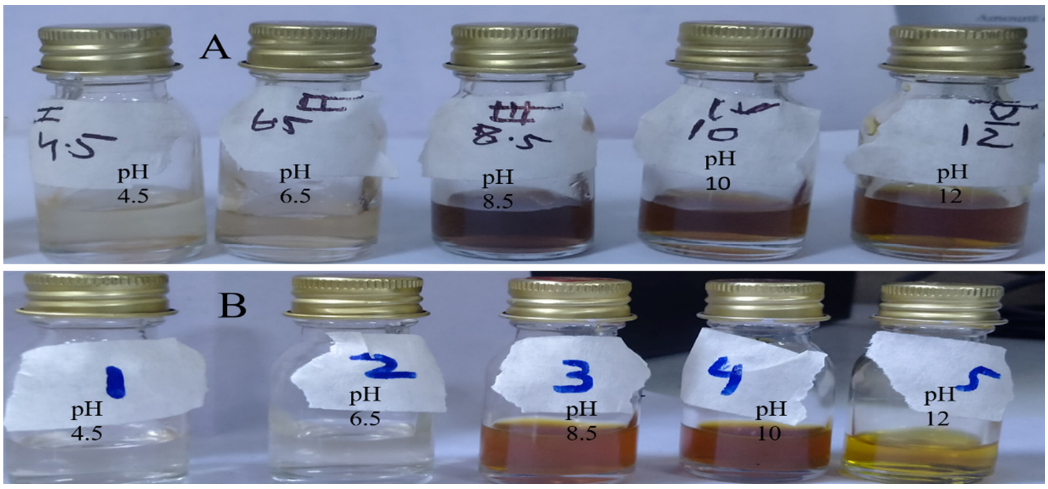

Figure S1. Visual observation of color changes at variation of pH after 24 hours biosynthesized silver nanoparticles using (**A**) aqueous extracts and (**B**) organic extracts of *Z. zerumbet*.

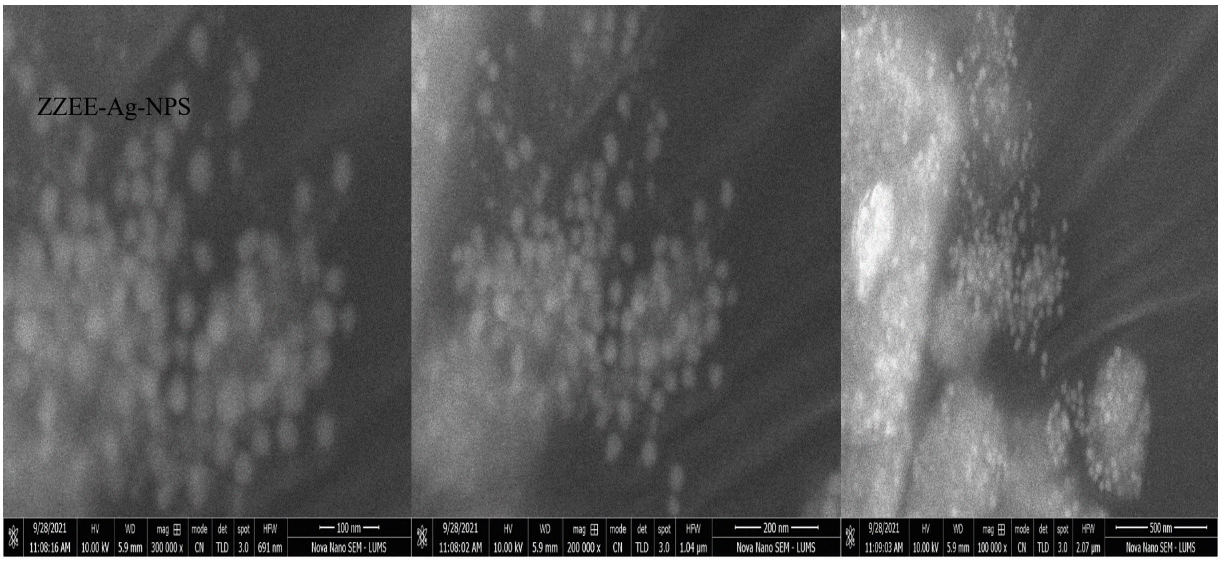

Figure S2. SEM micrograph showing the shape of AgNPs synthesized from ZZEE at different magnification.

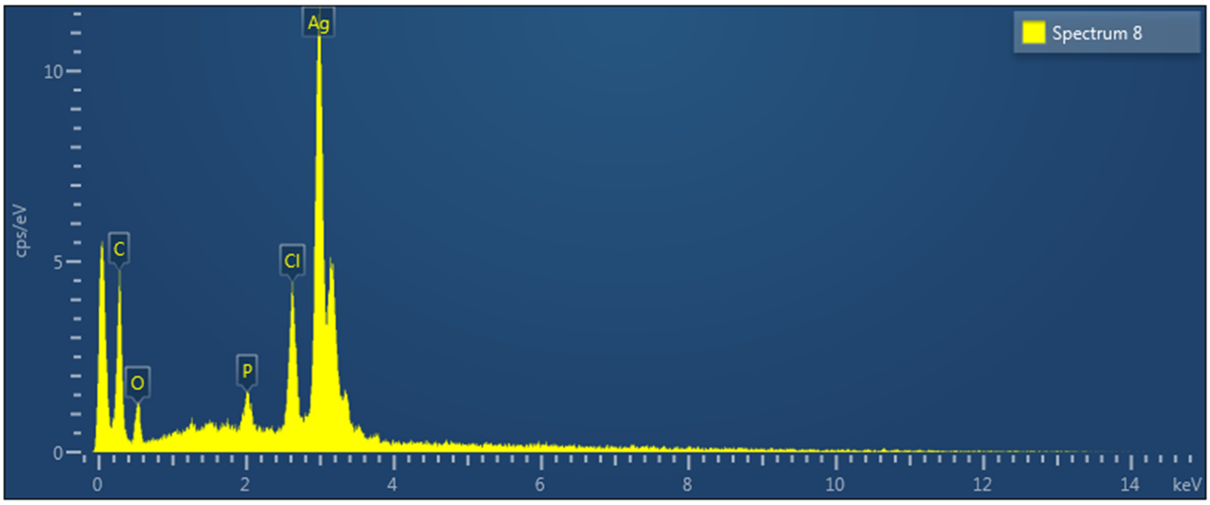

Figure S3. EDX spectra demonstrating the quantitative amount of different element in the synthesized silver nanoparticle using organic extract of *Z. zerumbet.*

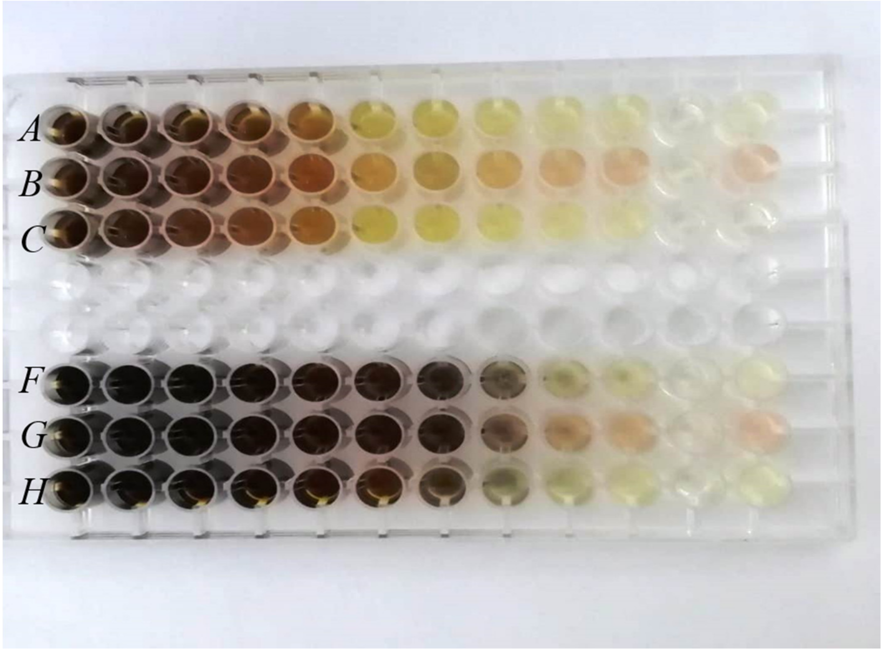

Figure S4. Micro-titer plates showing MIC top three row of silver nanoparticles using aqueous extract and bottom three rows of Ag-NPs using organic extract. Top Three rows A–C: *Staphylococcus aureus, Enterococcus faecalis* and *Streptococcus mutans,* the bottom three rows F–H: *Staphylococcus aureus, Enterococcus faecalis* and *Streptococcus mutans*. Columns 1-10: contain two-fold serial dilutions top three rows 50µg/ml ZZEE Ag-NPS and three bottom rows 50µg/ml synthesize ZZAE-Ag-NPs respectively. Column 11: NG (negative control well), Columns 12: PG (positive Control wells).

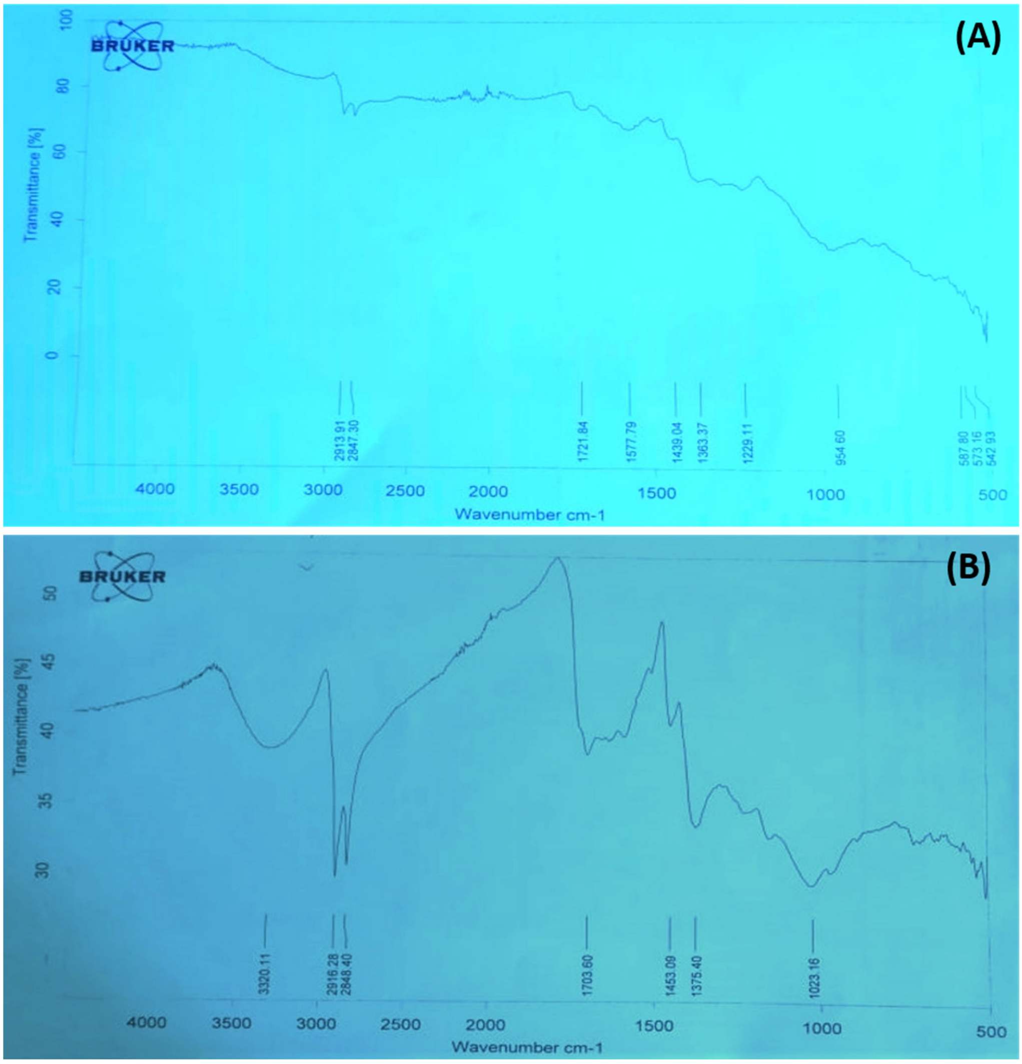

Figure S5. ATR- FTIR spectra (**A**) biosynthesized AgNPs using organic extract of *Z. zerumbet.* (**B**) biosynthesized AgNPs using aqueous extract of *Z. zerumbet*.

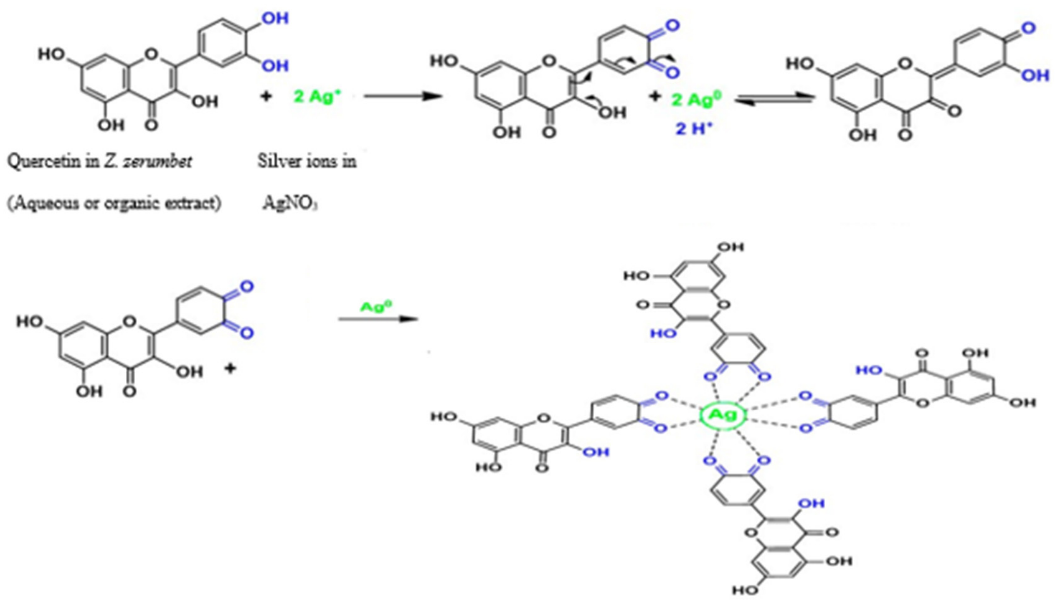

Figure S6. Mechanism for silver ion reduction to metallic silver nanoparticles via quercetin as reducing agent in *Z. zerumbet* Rhizome extract solution.

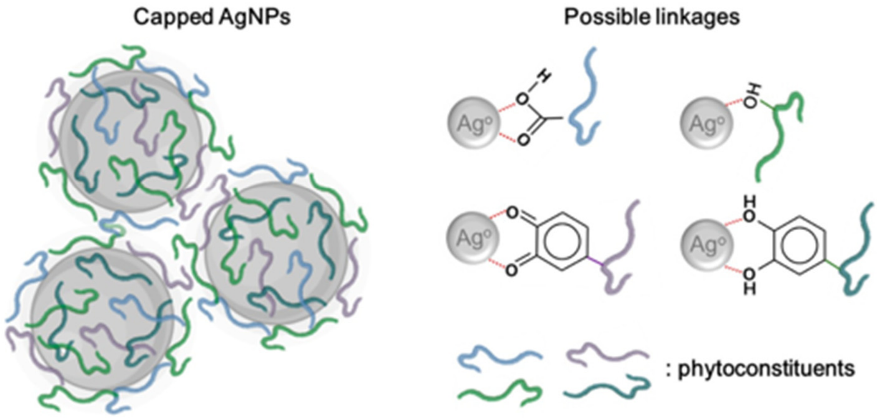

Figure S7. Schematic illustration of biosynthesized silver nanoparticle (ZZEE-Ag-NPs and ZZAE-Ag-NPs) Capped by phytoconstituents through carboxyl, hydroxyl and silver interactions.

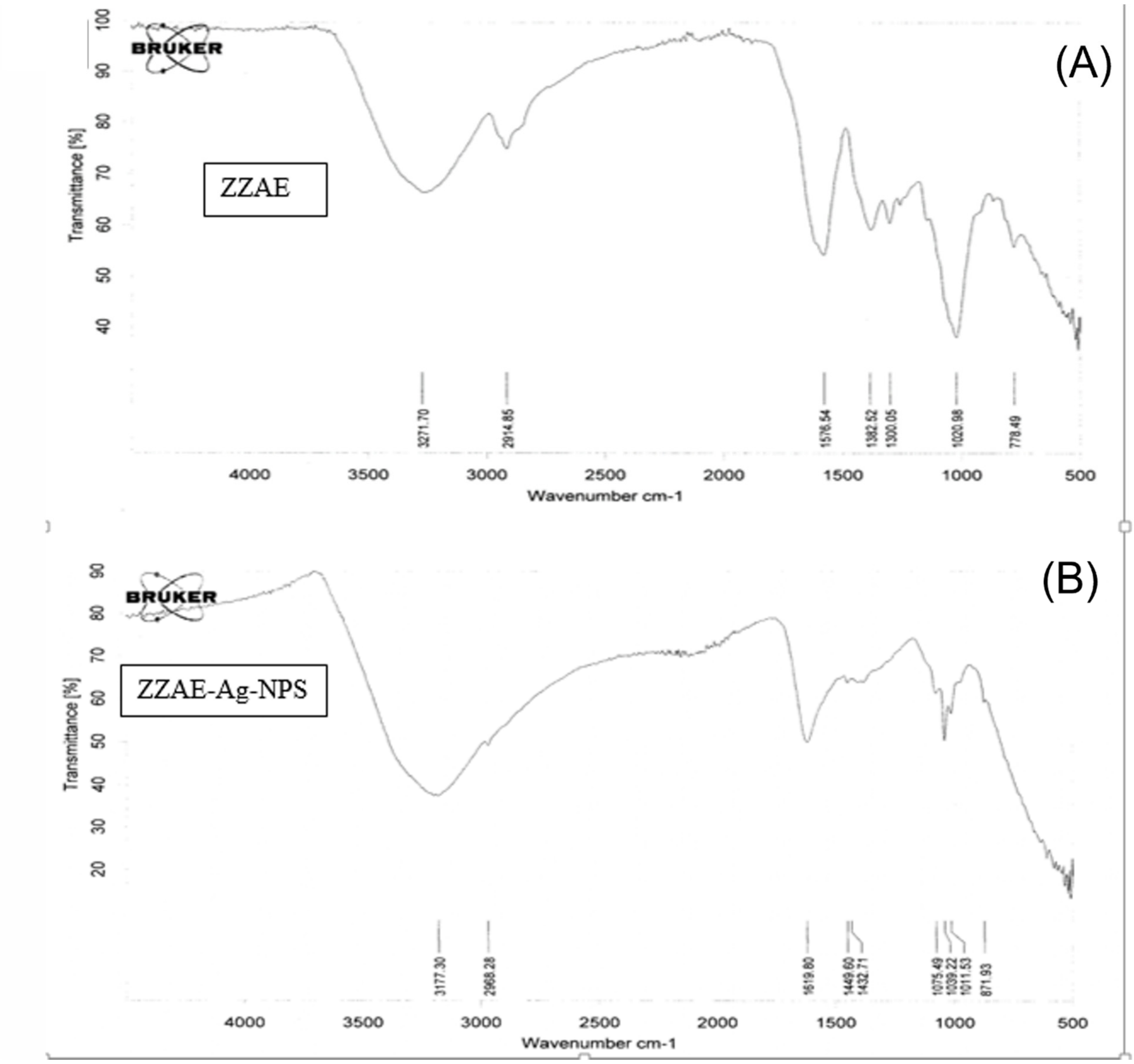

Figure S8. ATR-FTIR Spectra for (**A**) ZZEE and resultant ZZEE-Ag-NPs and (**B**) ZZAE and Their ZZAE-AG-NPs.

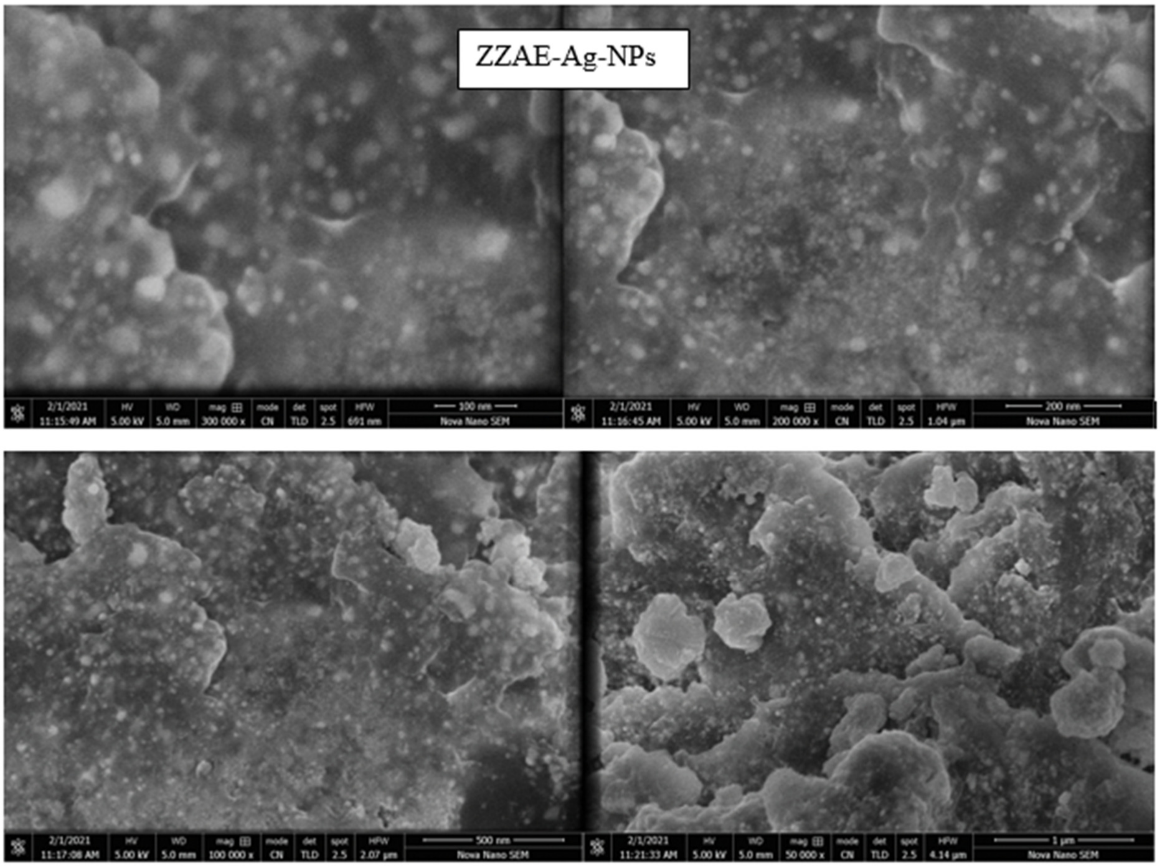

Figure S9. SEM micrograph showing the shape of AgNPs synthesized from ZZAE and ZZEE at different magnification.

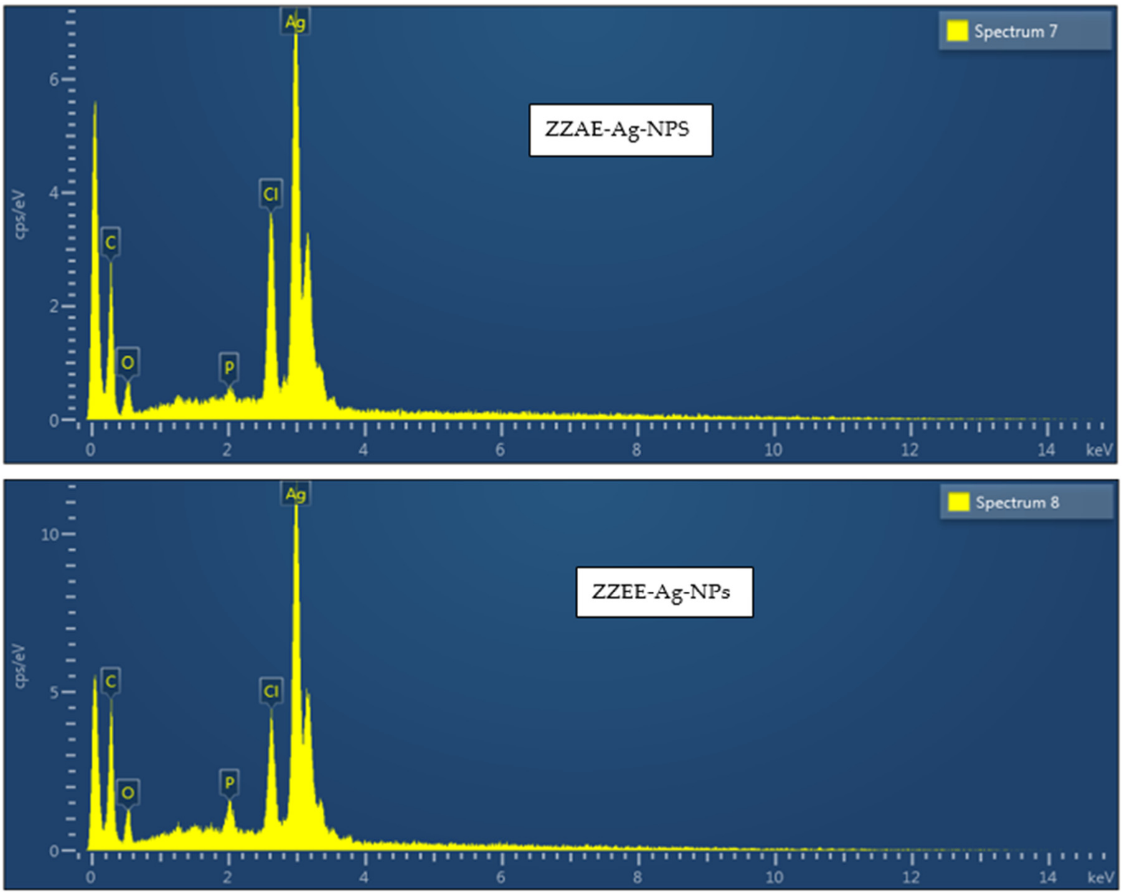

Figure S10. EDX spectra demonstrating the quantitative number of different elements in the synthesized silver nanoparticle using an aqueous and organic extract of *Z. zerumbet.*

Table S1. Antibacterial efficacy of commercial antibiotics against *E. coli, Salmonella enterica* and *S. aureus.*

Sr. No.	*Pathogens Species*	*Antibiotics*	*Zone of Inhibition (mm)*
1	*Enterococcus faecalis*	Amoxicillin	-
		Azocillin	-
		Cloxacillin	-
		Oxacillin	-
		Ticarcillin	-
2	*Streptococcus mutans*	Amoxicillin	-
		Azocillin	-
		Cloxacillin	-
		Oxacillin	-
		Ticarcillin	-
3	*S. aureus*	Amoxicillin	10
		Azocillin	-
		Cloxacillin	-
		Oxacillin	-
		Ticarcillin	-

Abbreviations: Ax_{25} (Amoxicillin), Az_{75} (Azocillin), Cx_{1} (Cloxacillin), Ox_{1} (Oxacillin), Ti_{75} (Ticarcillin).

Table S2. Abbreviation and their detail.

**Abbreviations**	**Detail**
AgNPs	Silver nanoparticles
ZZEE-AgNPs	Synthesized silver nanoparticles using organic or ethanol extract of *Z. zerumbet*
ZZAE-AgNPs	Synthesized silver nanoparticles using aqueous extract of *Z. zerumbet*
ZZAE	aqueous extract of *Z. zerumbet*
ZZAQE-AgNPs	Synthesized silver nano particles using aqueous extract of *Z. zerumbet*
ZZEE	Organic or ethanol extract of *Z. zerumbet*
Mg	Milligram
Ml	Milliliter
MIC	Minimum Inhibitory Concentration
MDR	Multidrug resistance
OD	Optical density
mM	Milli-mole
Rpm	Revolution per minutes
v/v	Volume by volume
w/v	Weight by volume
°C	Centigrade
